# Does repeated hydrodistension with transurethral fulguration for interstitial cystitis with Hunner’s lesion cause bladder contraction?

**DOI:** 10.1080/2090598X.2019.1589753

**Published:** 2019-04-08

**Authors:** Hikaru Tomoe, Kaori Yamashita

**Affiliations:** Department of Urology, Pelvic Reconstructive Surgery, Tokyo Women’s Medical University Medical Center East, Tokyo, Japan

**Keywords:** Interstitial cystitis, Hunner’s lesion, transurethral fulguration, bladder contraction

## Abstract

**Objective**: To evaluate the effect of repeated bladder hydrodistension with transurethral resection or fulguration (TUF) of Hunner’s lesions on bladder capacity and interstitial cystitis (IC) symptoms. TUF for Hunner’s lesion is recommended in several IC/BPS guidelines, although recurrence is highly probable.

**Patients and methods**: The study cohort comprised 44 IC patients with Hunner’s lesions who underwent multiple bladder hydrodistensions with TUF and electrocautery (EC) at our institution between July 2005 and June 2018. We analysed their frequency–volume chart, O’Leary Sant Interstitial Cystitis Symptom Index (ICSI), Interstitial Cystitis Problem Index (ICPI), and visual analogue scale (VAS) for pain, before and at 2, 6, and 12 months after surgery.

**Results**: The 44 Hunner-type IC patients underwent a total of 117 surgeries. Patients were divided into three groups based upon the number of hydrodistensions with TUF they underwent. Group 1 (*n* = 44), Group 2 (*n* = 44) and Group 3 (*n* = 21) underwent one, two, and three surgeries, respectively. At 6 months after surgery, the mean average voided volume (AVV) and maximum voided volume (MVV) were 157 mL and 228 mL in Group 1; 203 mL, 283 mL in Group 2; and 193 mL, 264 mL in Group 3. The AVV in Group 2 (P < 0.01) and Group 3 (P < 0.03), and the MVV in Group 2 (P < 0.02) increased significantly compared to Group 1. ICSI, ICPI and VAS pain score in groups 2 (P < 0.003, P < 0.01, P < 0.05) and 3 (P < 0.001, P < 0.001, P < 0.001) decreased significantly compared to Group 1.

**Conclusion**: Repeated hydrodistension and TUF with EC of Hunner’s lesions for recurrent Hunner-type IC patients improved their symptoms. There was a tendency toward an increase in bladder capacity and repeated hydrodistension with TUF did not appear to be a direct cause of bladder contraction.

**Abbreviations**: AVV: average voided volume; BPS: bladder pain syndrome; EC: electrical cautery; IC: interstitial cystitis; ICPI: Interstitial Cystitis Problem Index; ICSI: Interstitial Cystitis Symptom Index; MVV: maximum voided volume; TUF: transurethral fulguration; TUR: transurethral resection; VAS: visual analogue scale

## Introduction

The aetiology of interstitial cystitis (IC)/bladder pain syndrome (BPS) is unclear, and there is no curative treatment. At the Fourth International Consultation on Interstitial Cystitis Japan in 2018, it was suggested that IC/BPS be classified as IC with Hunner’s lesions, IC without Hunner’s lesions but with glomerulation, and BPS with no findings on cystoscopy. IC with Hunner’s lesions is considered to be a homogeneous disease, whereas IC/BPS with no Hunner’s lesions is considered to be a heterogeneous syndrome. Therefore, it is important to divide IC/BPS by type classification, as the response to treatment may differ depending on the type of IC/BPS.

For IC with Hunner’s lesions, transurethral fulguration (TUF) is recommended in the AUA Association IC/BPS Guidelines [1] and also is presented as a Grade B recommendation in the Asian Guidelines [2]. Both transurethral resection (TUR) [3–6] and TUF with electrocautery (EC) [7–9] or laser [10–12] have a noticeable effect, although recurrence is highly probable. Bladder perforation, haemorrhage, urethral stricture, VUR, small bowel adhesion to the bladder, and bowel injury, are known complications of these procedures. Furthermore, we often find bladder contraction in patients with IC who have received repeated surgeries during long-term follow-up. Some experts believe that repeated surgeries should be avoided in order to minimise the risk of bladder contraction. However, it is not clear whether bladder contraction occurs as a direct result of repeated TUR or TUF, or if it is related to the natural course of chronic inflammation due to IC. In our experience, repeated bladder hydrodistension with TUF with EC of Hunner’s lesions does not cause bladder contraction. In the present study, we evaluated the effects of repeated hydrodistension with TUF with EC of Hunner’s lesions in patients with Hunner-type IC.

## Patients and methods

We started bladder hydrodistension with TUF with EC of Hunner’s lesions for patients with Hunner-type IC in July 2005. The study cohort comprised 44 IC patients with Hunner’s lesion (42 women and two men) who underwent multiple hydrodistensions with TUF with EC at our institution between July 2005 and June 2018, and we analysed them retrospectively. Secondary IC caused by autoimmune diseases, such as Sjögren’s syndrome, was excluded. All patients were educated regarding self-care practices including dietary and behavioural modifications, such as stress avoidance before surgery. Hydrodistension was performed under spinal anaesthesia and the bladder was distended twice with normal saline at a pressure of 80 cmH_2_O for 3 min. Before hydrodistension, the bladder was inspected by cystoscopy for other potential symptom causes and for Hunner’s lesions. After hydrodistension, we coagulated the mucosal and submucosal layers of the Hunner’s lesions and the surrounding areas, with care taken to avoid coagulation into the muscle layer to minimise trauma to the muscle that could contribute to irreversible bladder contraction.

The maximum bladder capacity during hydrodistension was measured. Patients were asked to complete a 4-day frequency–volume chart, O’Leary Sant Interstitial Cystitis Symptom Index (ICSI), Interstitial Cystitis Problem Index (ICPI), and visual analogue scale (VAS) for pain, before and at 2, 6, and 12 months after surgery. We analysed these data retrospectively.

Statistical analyses were performed with JMP pro, version 13 (SAS Institute Inc., Cary, NC, USA). A *P* < 0.05 was considered statistically significant. The inter-group differences of characteristics were assessed using the Wilcoxon signed-rank test.

## Results

In all, 44 patients with Hunner-type IC underwent a total of 117 surgeries. The mean (SD) patient age at the first surgery was 66.8 (8.1) years. Depending on the number of surgeries that patients underwent, they were divided into three groups (Table 1). Patients in Group 1 (44 patients), Group 2 (44) and Group 3 (21) underwent one, two, and three surgeries, respectively. In other words, Group 1 shows the results of a first surgery; Group 2 shows the same people after a second surgery; and Group 3 shows the results of those patients who required a third surgery.

**Table 1.** Patients’ characteristics and preoperative data.

Variable	Group 1 (*n* = 44)	Group 2 (*n* = 44)	Group 3 (*n* = 22)	*P* Group 1 vs 2	*P* Group 1 vs 3
Gender, female:male, *n*	42:2	42:2	20:2		
Mean (SD):					
Age, years	66.8 (8.1)	68.8 (8.1)	72.3 (6.3)	0.125	0.002
24-h frequency, *n*	20.3 (14.5)	16.1 (6.4)	17.1 (8.7)	0.046	0.138
AVV, mL	102 (43)	117 (47)	123 (46)	0.056	0.039
MVV, mL	173 (74)	166 (63)	168 (70)	0.331	0.405
ICSI	15.8 (3.5)	13,2 (4.0)	12.0 (4.1)	0.001	<0.001
ICPI	12.9 (3.5)	10.0.(4.2)	9.9 (4.4)	<0.001	<0.005
VAS pain score	7.5 (2.1)	6.1 (2.3)	6.2 (2.4)	0.003	0.023
Bladder capacity at hydrodistention, mL	440 (133)	427 (148)	349 (131)	0.342	0.008

The differences between the two groups were analysed using the Wilcoxon signed-rank test.

The second surgery was performed at a mean (SD; median) of 25.4 (19.2; 20.5) months after the first and the third surgery was performed at a mean (SD; median) 28.0 (19.1; 20) months after the second. Eight patients underwent four surgeries, but they were not analysed due to the small cohort.

The patients’ characteristics and preoperative data are shown in Table 1. There was a significant difference between groups 1 and 2 (*P *< 0.05) in preoperative average 24-h urinary frequency. For the preoperative average voided volume (AVV) there was a significant difference between groups 1 and 3 (*P *< 0.04). The preoperative maximum voided volume (MVV) was not significantly different between the three groups. For preoperative ICSI, ICPI and VAS pain score, there was a significant difference between groups 2 (*P *< 0.001, *P *< 0.001, *P *< 0.002, respectively) and 3 (*P *< 0.001, *P *< 0.005, *P *< 0.02, respectively) and Group 1.

The mean (SD) maximum bladder capacity during hydrodistension of groups 1, 2 and 3 was 440 (133), 427 (148), 349 (131) mL, respectively. The maximum bladder capacity during hydrodistension of Group 3 was significantly smaller compared to groups 1 (*P *< 0.01) and 2 (*P *< 0.01).

**Figure 1.**
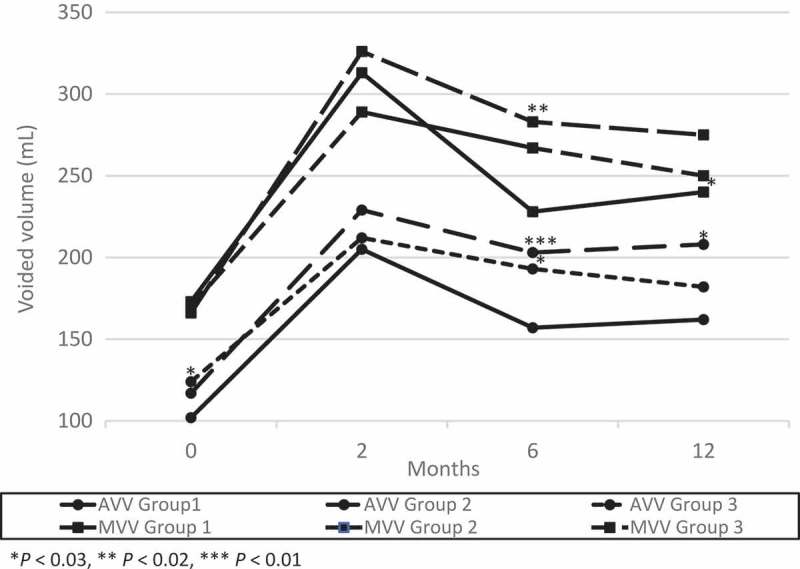
Changes from baseline in AVV and MVV. There was a significant difference between groups 2 or 3 and Group 1 in AVV at 6 months after surgery and the trend continued at 12 months after surgery.

**Figure 2.**
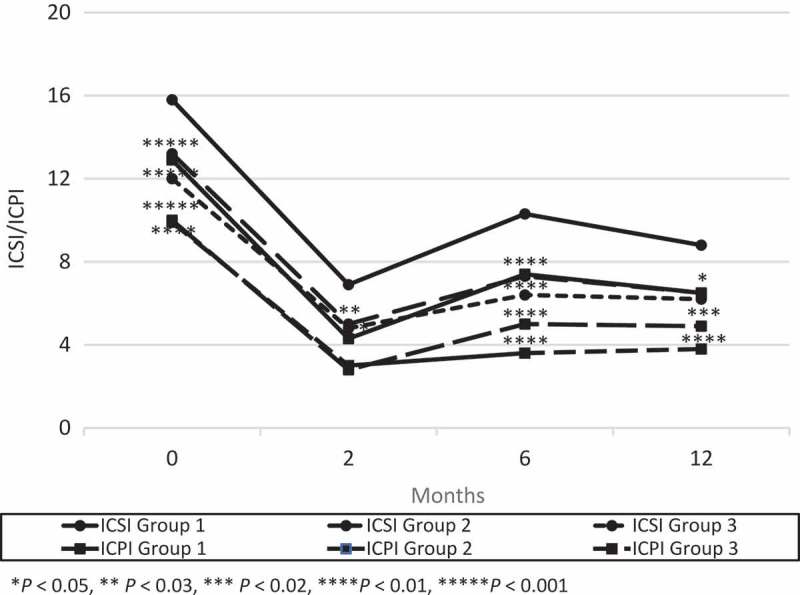
Changes from baseline in ICSI and ICPI. There was a significant difference between groups 2 or 3 and Group 1 in ICSI and ICPI at 6 months after surgery and the trend continued at 12 months after surgery.

**Figure 3.**
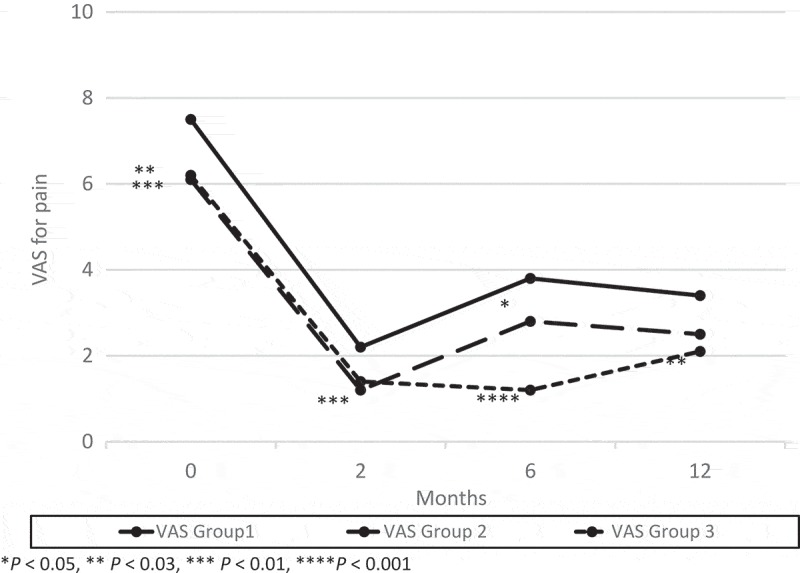
Changes from baseline in VAS for pain. There was a significant difference between groups 2 or 3 and Group 1 in VAS pain score at 6 months after surgery and the trend continued at 12 months after surgery.

At 2 months after their last surgery, there were no significant differences in frequency, AVV and MVV between the three groups (Figure 1). The mean (SD) ICSI, ICPI and VAS pain score were 6.9 (5.1), 4.3 (4.5), 2.1 (2.5) in Group 1; 5.0 (3.9), 2.8 (4.0), 1.2 (1.5) in Group 2; and 4.8 (3.8), 3.0 (4.0), 1.4 (2.4) in Group 3, respectively. The ICSI in groups 2 (*P *< 0.03) and 3 (*P *< 0.04), and VAS pain score in Group 2 (*P *< 0.01) significantly decreased compared to Group 1 (Figures 2 and 3).

Two of the 44 patients in Group 1 had worsened symptoms between 2 and 6 months, and received a second surgery. No patients in Group 2 showed recurrence between 2 and 6 months. At 6 months after surgery, there were no significant differences in frequency. The mean (SD) AVV and MVV were 157 (73) mL and 228 (106) mL in Group 1; 203 (102) mL and 283 (134) mL in Group 2; and 193 (60) mL and 264 (91) mL in Group 3, respectively. The AVV in Group 2 (*P* < 0.01) and Group 3 (*P* < 0.03), and the MVV in Group 2 (*P* < 0.02) increased significantly compared to Group 1 (Figure 1). The mean (SD) ICSI, ICPI and VAS pain score were 10.3 (4.7), 7.4 (4.5), and 3.8 (2.7) in Group 1; 7.3 (4.6), 5.0 (4.1) and 2.8 (2.7) in Group 2; and 6.3 (4.2), 3.4 (4.1) and 1.1 (1.9) in Group 3, respectively. The ICSI, ICPI and VAS pain score in groups 2 (*P* < 0.003, *P* < 0.01, *P* < 0.05) and 3 (*P* < 0.001, *P* < 0.001, *P* < 0.001) decreased significantly compared to Group 1 (Figures 2 and 3).

Seven of the 42 patients in Group 1 had worsened symptoms between 6 and 12 months, and received a second surgery. One of the 44 patients in Group 2 showed recurrence between 6 and 12 months, and received a third surgery. At 12 months after surgery, the mean (SD) AVV and MVV were 162 (82) mL and 240 (139) mL in Group 1; 208 (101) mL and 275 (135) mL in Group 2; and 183 (71) mL and 247 (84) mL in Group 3, respectively. There was a significant difference between Group 2 and Group 1 in the AVV (*P* < 0.03; Figure 1). The mean (SD) ICSI, ICPI and VAS pain score were 8.8 (5.0), 6.5 (4.5) and 3.4 (2.4) in Group 1; 6.5 (5.2), 4.9 (4.7) and 2.5 (2.7) in Group 2; and 6.4 (3.3), 4.1 (3.3) and 2.2 (2.4) in Group 3, respectively. The ICSI in groups 2 (*P* < 0.04) and 3 (*P* < 0.01), and ICPI in Group 3 (*P* < 0.004) decreased significantly compared to Group 1 (Figures 2 and 3).

## Discussion

Since the 1970s, TUR and TUF with EC for Hunner’s lesions have been reported to eliminate symptoms for ≥1 year in about half of patients [3–12]. They were also effective for patients with Hunner-type IC who were refractory to hydrodistension without TUF or had relapsed from the preceding TUR and showed a response to hydrodistension. The mechanism of action of TUR and TUF is speculated as removing inflamed nerve endings, reducing aggregates of potent inflammatory mediators and mast cell recruiting factors, and eliminating epithelial and sub-epithelial mast cells [13]. However, no significant correlation between mast cell density in the urothelium, lamina propria or detrusor, and duration of symptom amelioration could be seen after the first, second or third TUR [14].

To the best of our knowledge, there is no study that has estimated the effect of repeated bladder hydrodistension with TUF of Hunner’s lesions on bladder capacity and IC symptoms. In our present study, bladder hydrodistension with TUF with EC of Hunner’s lesions improved IC symptoms and tended to increase the bladder capacity in patients with recurrent Hunner-type IC.

Preoperative IC symptoms were significantly better in groups 2 and 3 compared to Group 1, which may only indicate that patients wanted re-operations before their symptoms became as severe as for their first surgery. This may be because the patients experienced symptomatic improvement after the initial surgery, and they had been observed for a long period of time until the first surgery due to a lack of diagnosis or conservative treatment.

The maximum bladder capacity during bladder hydrodistension in Group 3 was significantly smaller than in groups 1 and 2. This may be due to the fact that the number of patients in Group 3, which is highly likely to include many severe cases, was less than half of that of group 1 or 2. However, 24-h urinary frequency, AVV and MVV at 2 months after surgery significantly improved in all groups and there were no significant differences in them between the three groups. Furthermore, there was a significant difference between groups 2 and 3 and Group 1 in AVV, ICSI, ICPI and VAS pain score at 6 months after surgery and the trend continued at 12 months after surgery. This may suggest that a repeat bladder hydrodistension with TUF with EC of Hunner’s lesions does not cause bladder contraction. There is a possibility that superficial coagulation of Hunner’s lesions was successful, as that coagulation did not reach the muscle layer and muscle damage was avoided.

The present study is limited in its small sample size and its retrospective design. It is also non-randomised for ethical reasons as we cannot treat patients with Hunner-type IC by bladder hydrodistension alone without TUF, and we are also unable to coagulate both the mucosal and submucosal, and the muscle layer of the Hunner’s lesions.

## Conclusion

Repeated bladder hydrodistension and TUF with EC of Hunner’s lesions for recurrence in patients with Hunner-type IC contributed to an improvement in symptoms and did not reduce bladder capacity. Accordingly, the repeated surgeries appear highly unlikely to be a direct cause of bladder contraction. It is possible that excessively deep TUF may contribute to irreversible muscle layer damage that may lead to the contraction of the bladder.
